# If You Contemplate Buying an Automobile

**Published:** 1915-03

**Authors:** 


### If You Contemplate Buying an Automobile.

And have had no previous personal experience, it is worth while to consider the following:

1. Can you afford it? This does not mean can you get credit? Or can you mortgage something to raise the money? Or have you saved the price? But can you feel sure of an income that will justify the additional expense and leave enough for the necessities and luxuries that you want and which may be just as desirable as a means of transportation.

2. You can easily learn the price of various cars. Remember that this is merely an initiation fee, the annual dues varying from about half this price for very careful and moderate use, up to double the price for hard driving, inattention to details and bad luck. The actual mileage cost for the most economic automobile, run with the greatest economy, which means mainly daily attention to tires, use of spark and throttle, taking care of your own car, and anticipation of every defect that can cause trouble from neglect, can not possibly be brought below $2\frac{1}{2}$ cents on the average, not including insurance, garage rent, depreciation, repairs beyond those immediately depending on use. Counting original cost and replacement and current expenses, the permanent milage cost will come to 4 cents a mile at the very lowest estimate and from this minimum it will range up to 30 cents a mile for a fairly expensive car with chauffeur.

3. Do not imagine that the guarantee on an automobile or any supply is of the kind that you get from a department store, implying prompt, cheerful and free making good without regard to inevitable defects, carelessness, effects of wear, etc.

4. Do not imagine that the care of a car is easy and inexpensive. Roughly speaking, it will take half an hour of getting ready and adjusting, cleaning, etc., for every hour of running time. If you are of a mechanic turn of mind, and can mend clocks, put plumbing in order, do varnishing, adjust typewriters and sewing machines, you can probably learn in the course of a year or so to do similar work on the automobile, up to making major repairs and adjustments. You can easily calculate the actual value of your own time. Otherwise, estimate on the basis of plumbers' and electricians' wages.

5. Do not imagine that you can compare the mileage of an automobile to that of a horse and buggy. You will use the automobile much more and this is true economy as compared with various means of travel for pleasure but, remember also, the temptation to spend an excess of time simply in moving—for it is only exceptionally that an automobilist ever goes to a definite place for the pleasure and intellectual profit that that place affords; one glance at a waterfall, a hurried lunch in a grove, such glimpses of another city as can be caught at ten miles an hour, at most a card left at the door of a friend, and he is on the road again.

